# Acute Retinal Necrosis Caused by the Zoster Vaccine Virus

**DOI:** 10.1093/cid/cix683

**Published:** 2017-08-02

**Authors:** Gregory Heath, Daniel P Depledge, Julianne R Brown, Anthony D Hale, Helena Tutil, Rachel Williams, Judith Breuer

**Affiliations:** 1 Medical Ophthalmology, York Teaching Hospital NHS Foundation Trust, York, England; 2 Infection and Immunity, University College London, England; 3 Microbiology, Virology and Infection Control (VZV Typing Laboratory), Great Ormond St Hospital, London, England; 4 Virology, Leeds Teaching Hospitals NHS Trust, Leeds, England

**Keywords:** Varicella zoster virus, zoster, shingles, vaccine, retinitis

## Abstract

We report acute retinal necrosis caused by the vaccine Oka strain following immunization of a 78-year-old woman with live zoster vaccine. Whole genome sequencing confirmed the ocular vOka strain to be derived from the vaccine and excluded the presence of new mutations or recombination with wild-type Varicella zoster virus.

## CASE REPORT

A 78-year-old white female presented with a two-week history of floaters in her left eye. Her medical history was noteworthy for rheumatoid arthritis, latent autoimmune diabetes of adulthood (anti–glutamic acid decarboxylase antibody positive), and osteoporosis. Her medications included methotrexate 7.5 mg orally once a week, folic acid 5 mg daily (6 days per week), insulin (Humalog Mix 50/50 [insulin lispro protamine/insulin lispro] 30 units s/c), and alendronic acid orally 70 mg once a week. Her ophthalmic history was unremarkable. Six weeks before the onset of her ocular symptoms she had received the zoster vaccine (Zostavax).

**Figure 1.**
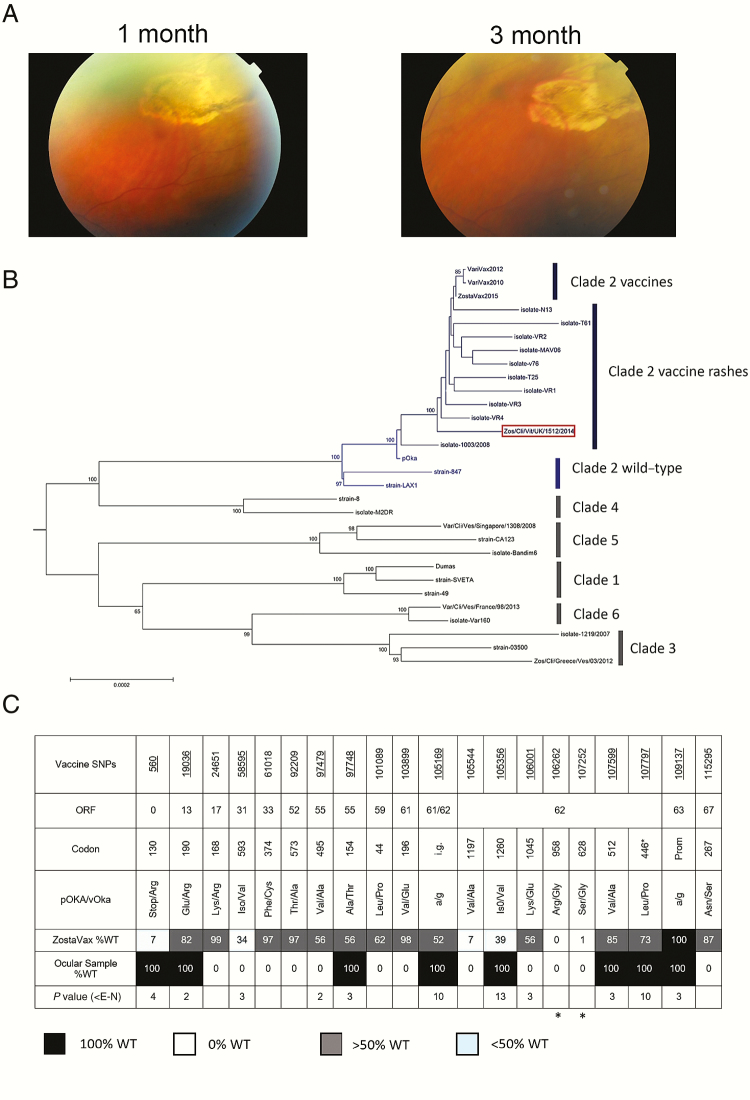
*A*, Healed acute retinal necrosis. *B*, Neighbor joining phylogenetic tree. Clades are colored as shown. The ocular strain is boxed. *C*, Schematic of key noncoding and nonsynonymous polymorphisms in the ocular vaccine strain compared with the zoster vaccine and the parental Oka strains. The first three rows show the vaccine nucleotides, open reading frame, and codon positions. The fourth row shows the amino-acid change in the vaccine. The fifth row shows the percentage of the wild-type nucleotide/amino acid found in the zostavax vaccine. The sixth row shows the sequence of the ocular vaccine strain, with vaccine mutations in white and wild-type mutations in black. Nucleotide positions at which the wild-type allele is selected for in vaccine rashes are underlined in row 1. The final row shows the strength of this selection (*P* value) for the wild-type nucleotide in vaccine rashes. The *s denote the 2 nonsynonymous fixed vaccine mutations detected by our real-time polymerase chain reaction. Abbreviations: ORF, open reading frame; pOKA, parental Oka; SNP, single nucleotide polymorphism; vOKA, vaccine Oka strain; WT, wild-type.

On examination, her visual acuities were 6 of 6 and 6 of 18 in her right and left eyes, respectively. Multiple, diffuse keratic precipitates were present in her left cornea. Although her left eye appeared white, there were cells (+++) in her anterior chamber. Her intraocular pressures were within the normal range at 14 mm Hg and 18 mm Hg in the right and left eyes, respectively. Fundus biomicroscopy confirmed cells (++) in her left vitreous cavity associated with haze (++). A white lesion in the superotemporal aspect of her left ocular fundus consistent with an area of retinitis was observed (Figure 1A).

Serum titers for toxoplasmosis, human immunodeficiency virus, and *Treponema pallidum* were negative. No history of varicella was obtained. Her full blood count and immunoglobulin levels were within the normal range. Her HbA1C was raised at 74 mmol/mol. She underwent a pars plana vitrectomy on the same day that she presented to analyze her vitreous fluid. Permission to report the findings was obtained.

## RESULTS

Cytological analysis of the sample of vitreous fluid revealed reactive lymphocytes. The fluid was positive for Varicella zoster virus DNA by real-time polymerse chain reaction (qPCR) and confirmed by genotype-specific qPCR [1] to be the vaccine Oka strain (vOka). Whole genome sequencing showed the virus to cluster phylogenetically with other vOka vaccine sequences (Figure 1B). There was no evidence of recombination with wild-type Varicella zoster virus. Compared with the sequence of the wild-type parental Oka (pOka) strain from which the vaccine was originally derived, the ocular vaccine strain had 12 vaccine mutations (Figure 1C), 10 of which have previously been observed in the vOka vaccine strain [2]. Positions K168R in open reading frame (ORF) 17 and F374C in ORF 33 have not previously been described in any vOka vaccine strain. However, deep sequencing of the zoster vaccine revealed these positions to be polymorphic at frequencies of 1% and 3%, respectively (Figure 1C). Vaccine viruses recovered from rashes and other postimmunization complications have fewer vaccine mutations than vOka viruses found in the vaccine preparation itself. In particular, selection for the ancestral pOka allele is observed at >2 of 11 specific loci [3], 8 of which were wild-type in the ocular vaccine virus sequenced here (Figure 1C).

The patient received oral valaciclovir 2 g 3 times a day for 3 weeks, reducing down to 1 g three times a day for a total period of 3 months before stopping altogether. In addition, she was administered a tapering dose of topical prednisolone acetate 1% for 4 weeks and atropine 1% into her left eye to address her secondary anterior uveitis. Her visual acuity improved from 6 of 18 to 6 of 9 within 2 weeks. The area of retinitis healed, leaving a pigmented scar (Figure 1A).

## DISCUSSION

This patient met the diagnosis of acute retinal necrosis, which was defined by the American Uveitis Society [4] as >1 foci of retinal necrosis, progression in the absence of antiviral therapy, an occlusive vasculopathy with arterial involvement, and a prominent inflammatory reaction in both the anterior and vitreous chambers. Acute retinal necrosis is a rare ophthalmic disease with an incidence of 0.63 per million population per year in the United Kingdom [5]. Although classically described in immunocompetent patients, immunodeficiency is observed in at least 30% of cases and is associated with more severe disease [6]. Presentation appears to be bimodal and is dependent on the underlying etiology. Acute retinal necrosis secondary to herpes simplex virus type 2 occurs at a mean age of 27 years, whereas cases occurring secondary to herpes simplex virus type 1 or herpes zoster affect older ages (mean age, 58 years) [6].

Although the use of intravenous aciclovir has been regarded as the standard treatment, currently oral valaciclovir or valganciclovir are recommended for treatment of acute retinal necrosis secondary to herpes simplex/zoster and cytomegalovirus, respectively [7]. An oral dose of valaciclovir 2 g three times a day has been shown to achieve systemic levels comparable with intravenous aciclovir [7]. For those patients with aciclovir resistance or in whom their retinitis threatens/involves their macula or optic nerve, intravitreal foscarnet may be injected into the affected eye twice or thrice weekly [7]. Oral corticosteroids may be added for patients with severe inflammation or sight-threatening disease. Topical corticosteroids combined with cycloplegia is often prescribed to ameliorate anterior segment inflammation. In line with recent recommendations, aspirin was not prescribed [7].

Herpes zoster is a potentially devastating disease affecting >30% of those aged >70 years, with serious complications, mainly prolonged debilitating pain occurring in 50%. The Zostavax vaccine, which contains the live attenuated vOka strain of varicella zoster virus, has been shown to reduce the incidence of shingles and post herpetic neuralgia by 51.3% and 66.5%, respectively [8]. Immunization of adults aged ≥70 years began in the United Kingdom in 2013. Coverage reached 60%, within 2 years, and this has already resulted in significant falls in the incidence of zoster in vaccinated cohorts (G. Amirthalingam, personal communication). Because the vaccine contains a live attenuated strain, its use in patients who are immunosuppressed is contraindicated. Notwithstanding, current advice is that immunization is safe for patients receiving low-doses of methotrexate (<0.4 mg/kg/wk), azathioprine (<3.0 mg/kg/d), or 6 mercaptopurine (<1.5 mg/kg/d) for the treatment of autoimmune and inflammatory diseases [9]. Despite many hundreds of thousands of patients having received the vaccine, complications resulting from replication of the vaccine virus have rarely been reported [10]. One possible but unconfirmed vOka rash occurring within 6 weeks of immunization was reported in clinical trials of >60000 individuals [10, 11]. A second fatal case of vOka vaccine strain dissemination has also been reported in an immunocompromised patient in whom the shingles vaccine was contraindicated [1]. A single case of vOka herpes zoster following the shingles vaccine has been reported [12], but there have been no cases of acute retinal necrosis confirmed as due to the vOka vaccine strain in a patient deemed suitable to receive the zoster vaccine. This case illustrates, however, the need to consider a vaccine etiology in patients who present with unusual symptoms and have received the zoster vaccine.

We were able to recover and sequence the whole viral genome from the vitreous fluid. From this we proved that no recombination between the vaccine strain and the patient’s autochthonous wild-type virus had occurred, something that, although theoretically possible, has to date not been described for any vOka vaccine strain. We show that all 12 vaccine mutations, including 2 apparently new mutations in ORFs 17 and 33, neither of which lies within domains known or predicted to affect function of these proteins, are also present in the zoster vaccine preparation, thus confirming that no new mutations had occurred (Figure 1C). Despite the vaccine containing many mixed positions (fifth row down, Figure 1C,) the ocular virus was monomorphic (ie, single nucleotide polymorphisms were either 100% vaccine [white cell] or 100% wild-type [black cell] [sixth row, Figure 1C]), suggesting that it is likely to have arisen from infection by a single virion that spread hematogenously after immunization. Whether this strain is more virulent is unclear. However, the ocular vOka strain had the genetic characteristics that have previously been demonstrated to be associated with increased likelihood of rash formation and other postimmunization complications [3]. In particular, the wild-type (pOka) amino acid (black cell) was present in the ocular virus at 3 positions in IE62 that have been most strongly associated with rash formation (*P* < 10^−10^) (Figure 1C). This includes the leucine at position 446 in the ORF62 major transactivating protein, which despite being mixed in vOka vaccine preparations, is always wild-type in rashes and other complications, implying a critical role for in vivo replication of the vaccine strain.

In summary, we report a case of retinitis, caused by the vOka strain following zoster vaccine, which responded well to antiviral treatment. Although very few complications caused by replication of the attenuated vOka strain have been reported following the zoster vaccine, this case illustrates the need to investigate unusual presentations occurring in recently immunized individuals.

## Notes

***Acknowledgments.*** We acknowledge the support of the UK Medical Research Council (MRC)/National Institute for Health Research (NIHR) University College London (UCL) Pathogen Genomics Unit. All research at Great Ormond Street Hospital NHS Foundation Trust and UCL Great Ormond Street Institute of Child Health is made possible by the NIHR Great Ormond Street Hospital Biomedical Research Centre.

***Disclaimer.*** The views expressed are those of the author(s) and not necessarily those of the National Health Service (NHS), the NIHR, or the Department of Health.

***Financial support.*** This work was supported by a New Investigator Award from the Medical Research Foundation (UK MRC); NIHR funding (grant NIHR-HCS-D12-03-15 to D. P. D.); and NIHR UCL/UCLH Biomedical Research Consortium funding (to J. B.).

***Potential conflicts of interest.*** All authors: No reported conflicts of interest. All authors have submitted the ICMJE Form for Disclosure of Potential Conflicts of Interest. Conflicts that the editors consider relevant to the content of the manuscript have been disclosed.
